# Differential diagnosis of breast mucinous carcinoma with an oval shape from fibroadenoma based on ultrasonographic features

**DOI:** 10.1186/s12905-024-02910-w

**Published:** 2024-02-03

**Authors:** Hongli Wang, Yue Hu, Cui Tan, Ran Gu, Yudong Li, Liang Jin, Xiaofang Jiang, Jingsi Mei, Qiang Liu, Chang Gong

**Affiliations:** 1 Guangdong Provincial Key Laboratory of Malignant Tumour Epigenetics and Gene Regulation, Sun Yat-Sen Memorial Hospital, Sun Yat-Sen University, Guangzhou, 510120 China; 2 Breast Tumor Center, Sun Yat-Sen Memorial Hospital, Sun Yat-Sen University, Guangzhou, 510120 China; 3 Department of Pathology, Sun Yat-Sen Memorial Hospital, Sun Yat-Sen University, Guangzhou, 510120 China

**Keywords:** Breast, Ultrasonography, Mucinous carcinoma

## Abstract

**Background:**

Approximately 50% of breast mucinous carcinomas (MCs) are oval and have the possibility of being misdiagnosed as fibroadenomas (FAs). We aimed to identify the key features that can help differentiate breast MC with an oval shape from FA on ultrasonography (US).

**Methods:**

Seventy-six MCs from 71 consecutive patients and 50 FAs with an oval shape from 50 consecutive patients were included in our study. All lesions pathologically diagnosed. According to the Breast Imaging Reporting and Data System (BI-RADS), first, the ultrasonographic features of the MCs and FAs were recorded and a final category was assessed. Then, the differences in ultrasonographic characteristics between category 4 A (low-risk group) and category 4B-5 (medium-high- risk group) MCs were identified. Finally, other ultrasonographic features of MC and FA both with an oval shape were compared to determine the key factors for differential diagnosis. The Mann-Whitney test, *χ*^*2*^ test or Fisher’s exact test was used to compare data between groups.

**Results:**

MCs with an oval shape (81.2%) and a circumscribed margin (25%) on US were more commonly assessed in the low-risk group (BI-RADS 4 A) than in the medium-high-risk group (BI-RADS 4B-5) *(*20%, *p* < 0.001 and 0%, *p* = 0.001, respectively). Compared with those with FA, patients with MC were older, and tended to have masses with non-hypoechoic patterns, not circumscribed margins, and a posterior echo enhancement on US (*p* < 0.001, *p* < 0.001, and *p* = 0.003, respectively).

**Conclusion:**

The oval shape was the main reason for the underestimation of MCs. On US, an oval mass found in the breast of women of older age with non-hypoechoic patterns, not circumscribed margins, and a posterior echo enhancement was associated with an increased risk of being an MC, and should be subjected to active biopsy.

## Background

Breast mucinous carcinoma (MC) is characterized by clusters of generally small and uniform cells floating in large amounts of extracellular mucin. It is a rare histological type with a prevalence of 1–7% among all breast carcinomas [1]. MC grows slowly, has a low rate of lymph nodal metastasis, and has a good prognosis [2, 3].

At present, ultrasonography (US) is one of the most widely available diagnostic options for women with breast mass [4]. The descriptions of ultrasonographic features of breast masses are mainly based on the Breast Imaging Reporting and Data System (BI-RADS) [5]. Morphological feature is an important factor in the differential diagnosis of benign and malignant masses [6, 7]. In general, an irregular shape is highly suggestive of malignancy, while an oval shape indicates benignity [6, 7]. However, as a slow-growing carcinoma, approximately 50% of MCs were oval shape [8]. Due to the relatively high frequency of MC with an oval shape, there is potential for misdiagnosing it as fibroadenoma (FA), which is the most common benign breast solid mass.

Bode et al. [9] reported that 92% of MCs could be classified as BI-RADS category 4 or above on US. However, the malignant ultrasonographic features of MCs are more subtle than those of typical breast carcinoma, and therefore some of them may be assessed as BI-RADS category 4 A. Compared with category 4B and above, the malignant likelihood of category 4 A lesions is low between 2% and 10%, which belong to low-risk. Furthermore, among category 4 A masses, the likelihood of malignancy is lower for those with an oval shape than for those with an irregular shape, even less than 2% [10]. Follow-up instead of biopsy is advised for the category 4 A masses with an oval shape, which may be MCs [10, 11]. Thus, MCs that were assessed as category 4 A may have a delayed diagnosis.

Therefore, first, this retrospective study explored the ultrasonographic features that lead to the assessment of MCs as BI-RADS category 4 A (low-risk group) by comparing them with BI-RADS category 4B and above MCs (medium-high-risk group). Second, the specific ultrasonographic features that can help differentiate MC with an oval shape from FA were identified.

## Methods

### Patients

**Fig. 1:**
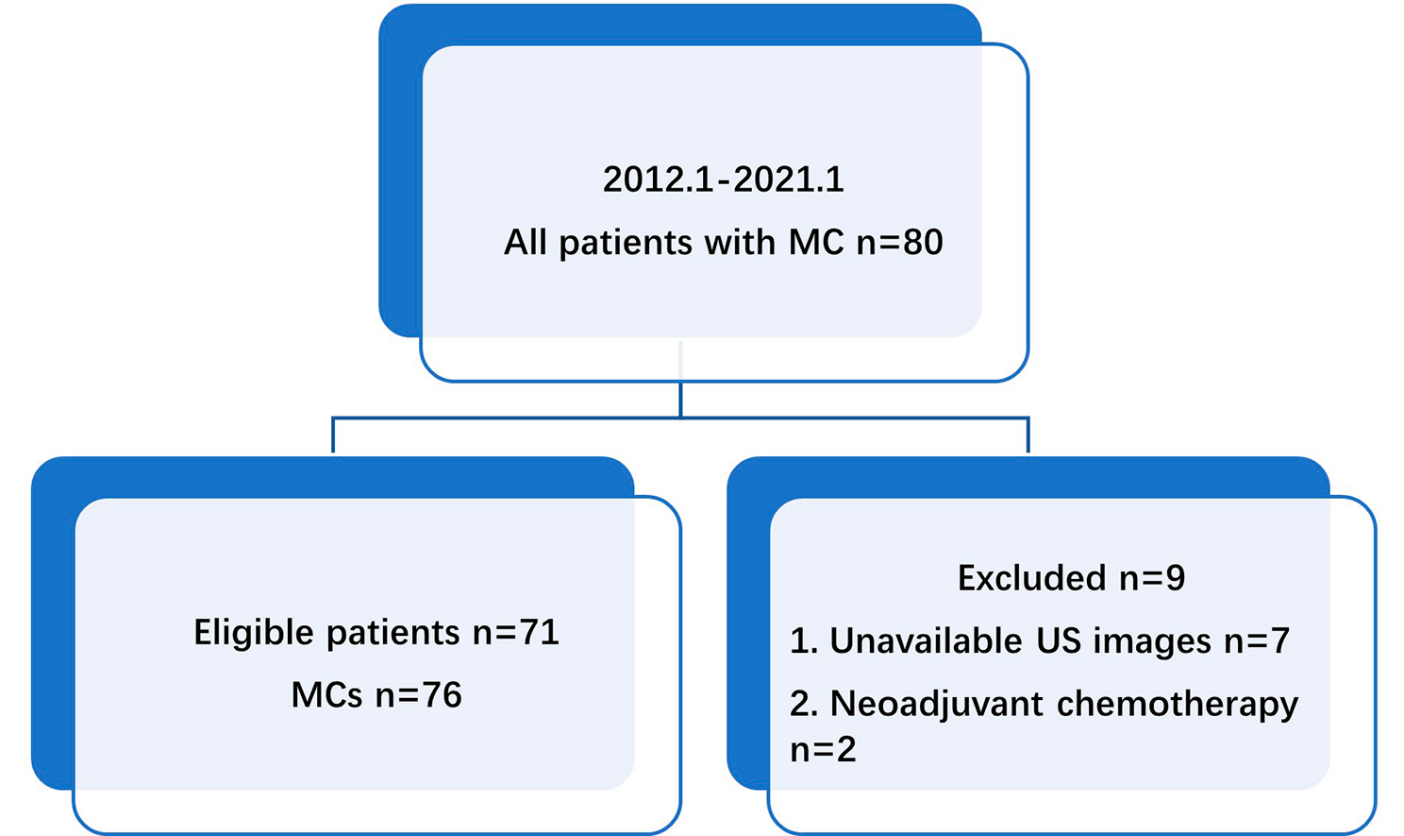
Flow chart of the selection process for mucinous carcinoma (MC) patients

The pathology database of our hospital was searched from January 2012 to January 2021 to identify surgically treated MC patients. Depending on the proportion of MC components, the MCs were separated into two pathologic types: pure mucinous carcinoma (PMC, ≥ 90% mucinous carcinoma) and mixed mucinous carcinoma (MMC, < 90% mucinous carcinoma). The inclusion criteria for patients with MC were as follows: (1) surgical excision of the breast MCs; (2) breast US before biopsy in our hospital; (3) no history of breast cancer. The exclusion criteria were as follows: (1) unavailable US images; (2) neoadjuvant chemotherapy. Consequently, 76 MCs in 71 consecutive patients (two patients had three MCs each, and one patient had two MCs) (Fig. 1) were selected for retrospective analysis. of these, 49 (64.5%) were PMCs, while 27 (35.5%) were MMCs.

In addition, the pathology database was searched for FA patients admitted between June 2019 and January 2021. The inclusion and exclusion criteria for patients with FA were the same as those for patients with MC, but the shape of the FA was limited to oval. If the patients had multiple FAs in the unilateral breast, the largest FA was included in our study. Finally, 50 FAs in 50 consecutive patients were selected for the research.

### Ultrasonography

Breast US examinations were performed with a high-resolution US unit (S2000, S1000 or ACUSON Oxana2; Siemens Medical Solutions, Erlangen, Germany). During the examination, the patients were in the supine position with their arms raised and hands placed behind their heads.

All US scans were performed and interpreted simultaneously by one dedicated breast radiologist with a minimum of 5 years of experience. The age of the patients [12] and the following features of MCs and FAs according to the fifth edition of the BI-RADS guidelines [5] were evaluated: maximum dimension (cm), shape (oval, irregular), orientation (parallel, not parallel), margin (circumscribed, not circumscribed), echo pattern (hypoechoic, isoechoic, heterogeneous and complex cystic and solid), posterior features (enhancement, no posterior features), calcifications in the mass (yes, no) and vascularity (absent, internal vascularity and vessels in rim). Because of the small number of cases in this study, isoechoic, heterogeneous and complex cystic and solid echo patterns were classified as non-hypoechoic pattern. Then, a final category was assessed. All MCs were assessed as category 4 A and above. Furthermore, MCs were divided into a low-risk group (category 4 A) and a medium-high-risk group (categories 4B-5). Consequently, 16 MCs were classified as the low-risk group and 60 were classified as the medium-high-risk group. Thirty-nine FAs were classified as category 4 A and 11 were classified as category 3. According to the BI-RADS guideline, category 3 lesions only require six months of follow-up, and no biopsy is needed. However, the 11 patients with category 3 FAs strongly requested and were granted biopsy.

### Statistical analysis

Age and maximum dimension were compared between groups using the Mann-Whitney test. The *χ*^*2*^ test or Fisher’s exact test was used to compare the ultrasonographic features of the low-risk and medium-high-risk groups of MCs as well as those of FAs versus MCs both with oval shapes on US. SPSS 20.0 (IBM, Chicago, IL, USA) was used for the statistical analyses. All *P* values were two-tailed, and *P* < 0.05 was considered to indicate statistical significance.

## Results

### Patient and ultrasonographic features of MC

The median age of the 71 patients with MC was 47 years (range: 27–86 years). 76 MCs presented as masses on US. The median maximum dimension was 2.5 cm (range: 1.0–8.0 cm). The ultrasonographic features of the MCs are shown in Table 1. No correlations were found between the ultrasonographic features and pathologic types.

**Table 1:** Ultrasonographic features of mucinous carcinomas

Features	MC	PMC	MMC	*P* value
Age	47 (41∽61)	47 (42∽57)	45 (39∽65)	0.757
Maximum dimension	2.5 (1.7∽3.2)	2.5 (1.7∽3.4)	2.4 (1.8∽3.0)	0.879
Shape				0.653
Oval	25 (32.9%)	17 (34.7%)	8 (29.6%)	
Irregular	51 (67.1%)	32 (65.3%)	19 (70.4%)	
Orientation				0.338
Parallel	65 (85.5%)	40 (81.6%)	25 (92.6%)	
Not parallel	11 (14.5%)	9 (18.4%)	2 (7.4%)	
Margin				0.323
Circumscribed	4 (5.3%)	4 (8.2%)	0 (0.0%)	
Not circumscribed	72 (94.7%)	45 (91.8%)	27 (100.0%)	
Echo				0.445
Hypoechoic	33 (43.4%)	21 (42.9%)	12 (44.5%)	
Isoechoic	25 (32.9%)	14 (28.5%)	11 (40.7%)	
Heterogeneous	16 (21.1%)	12 (24.5%)	4 (14.8%)	
Complex cystic and solid	2 (2.6%)	2 (4.1%)	0 (0.0%)	
Posterior features				0.838
No posterior features	27 (35.5%)	17 (34.7%)	10 (37.0%)	
Enhancement	49 (64.5%)	32 (65.3%)	17 (63.0%)	
Calcifications in the mass				0.822
No	67 (88.2%)	44 (89.8%)	23 (85.2%)	
Yes	9 (11.8%)	5 (10.2%)	4 (14.8%)	
Vascularity				0.161
Absent	15 (19.7%)	12 (24.5%)	3 (11.1%)	
Internal vascularity or vessels in rim	61 (80.3%)	37 (75.5%)	24 (88.9%)	

Abbreviations: MC: mucinous carcinoma; PMC: pure mucinous carcinoma; MMC: mixed mucinous carcinoma

### Differences in ultrasonographic features of MC between the low-risk group and medium-high-risk group

According to the BI-RADS, the MCs were divided into a low-risk group (BI-RADS category 4 A) and a medium-high-risk group (BI-RADS category 4B-5). The differences in the shape and margin between the two groups were statistically significant (*p* < 0.001, *p* = 0.001, respectively) (Table 2), especially the former. A total of 81.2% MCs in the low-risk group but only 20.0% in the medium-high-risk group were oval. Moreover, all of the MCs in the medium-high-risk group had not circumscribed margins versus only 75% of MCs in the low-risk group.

**Table 2:** Differences in ultrasonographic features between the low-risk group and the medium-high-risk group of mucinous carcinomas

Feature	Low-risk group	medium-high risk group	*P* value
Age	48 (42∽60)	46 (40∽62)	0.610
Maximum dimension	2.1(1.5∽2.8)	2.5 (1.8∽3.5)	0.096
Shape			< 0.001
Oval	13(81.2%)	12(20.0%)	
Irregular	3(18.8%)	48(80.0%)	
Orientation			1.000
Parallel	14(87.5%)	51(85.0%)	
Not parallel	2(12.5%)	9(15.0%)	
Margin			0.001
Circumscribed	4(25.0%)	0(0.0%)	
Not circumscribed	12(75.0%)	60(100.0%)	
Echo			0.976
Hypoechoic	7(43.8%)	26(43.3%)	
Non-hypoechoic	9(56.2%)	34(56.7%)	
Posterior features			0.115
No posterior features	3(18.8%)	24(40.0%)	
Enhancement	13(81.2%)	36(60.0%)	
Calcifications in the mass			0.225
No	16(100%)	51(85.0%)	
Yes	0(0%)	9(15.0%)	
Vascularity			0.098
Absent	6(37.5%)	9(15.0%)	
Internal vascularity and vessels in rim	10(62.5%)	51(85.0%)	

### Comparison of ultrasonographic features between MC with an oval shape and FA with an oval shape

**Fig. 2:**
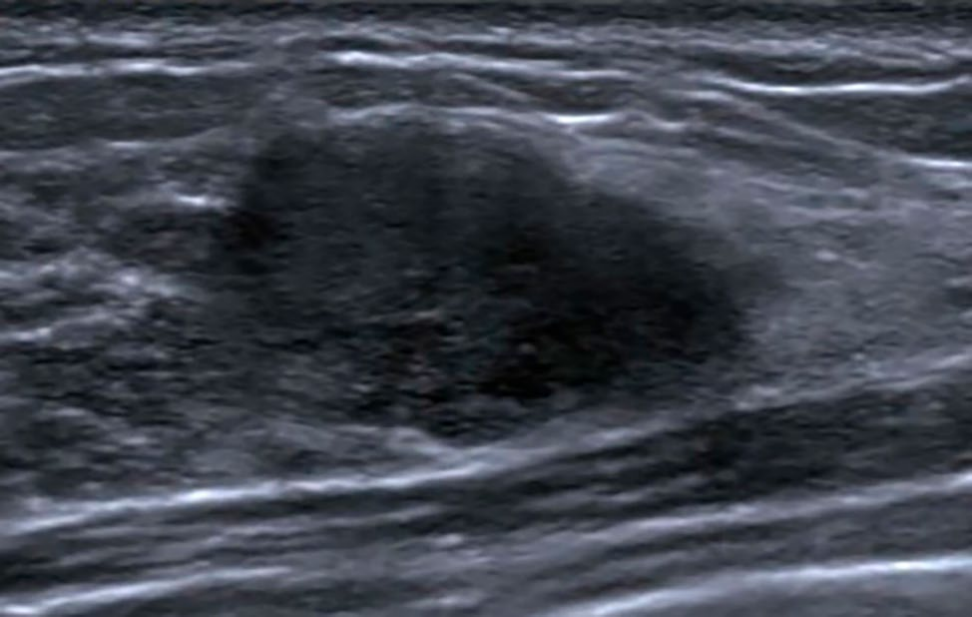
Fibroadenoma in a 34-year-old woman. An oval mass with a hypoechoic pattern, not circumscribed margins and no posterior features was found in the right breast. The mass was categorized as 4 A mainly because of the not circumscribed margin. Pathological diagnosis: fibroadenoma

**Fig. 3:**
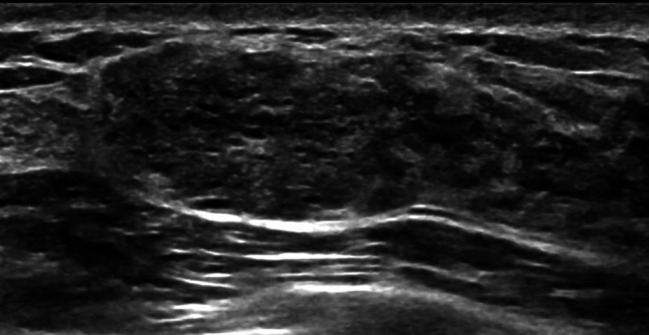
Mucinous carcinoma in a 45-year-old woman. An oval mass with non-hypoechoic patterns, not circumscribed margins and a posterior echo enhancement was found in the right breast. The mass was categorized as BI-RADS 4 A mainly due to the not circumscribed margin. Pathological diagnosis: mucinous carcinoma

Next, the other ultrasonographic features of MC and FA both with an oval shape were compared. The differences in patient age, ultrasonographic margins, internal echo patterns, and posterior features between the two groups were statistically significant (*p* < 0.001, *p* < 0.001, *p* < 0.001, *p* = 0.003, respectively) (Table 3). Compared to those with FAs (Fig. 2), the patients with MCs were older and more likely to have MCs with not circumscribed margins, non-hypoechoic patterns (including isoechoic, heterogeneous, complex cystic and solid echo), and with a posterior echo enhancement (Fig. 3).

**Table 3:** Comparison of other ultrasonographic features between MC with an oval shape and FA with oval shape

Features	MC	FA	*P* value
Age	47 (42∽56)	30 (24∽36)	< 0.001
Maximum dimension	2.3 (1.4∽3.3)	2.1 (1.4∽2.6)	0.306
Orientation			0.069
Parallel	22(88.0%)	49(98.0%)	
Not parallel	3(12.0%)	1(2.0%)	
Margin			< 0.001
Circumscribed	4(16.0%)	42(84.0%)	
Not circumscribed	21(84.0%)	8(16.0%)	
Echo			< 0.001
Hypoechoic	9(36.0%)	49(98.0%)	
Non-hypoechoic	16(64.0%)	1(2.0%)	
Posterior features			0.003
No posterior features	6(24.0%)	30(60.0%)	
Enhancement	19(76.0%)	20(40.0%)	
Calcifications in the mass			1.000
No	24(96.0%)	48(96.0%)	
Yes	1(4.0%)	2(4.0%)	
Vascularity			0.307
Absent	7(28.0%)	20(40.0%)	
Internal vascularity and vessels in rim	18(72.0%)	30(60.0%)	

Abbreviations: FA: fibroadenoma; MC: mucinous carcinoma

## Discussion

Breast MC is rare in clinical practice, and a preoperative diagnosis is difficult to obtain due to the variability in ultrasonographic features and the overlap of its morphological features with those of benign tumours reported in the literature [8, 9, 13–17].

In our study, although most MCs showed malignant signs and were assessed as being medium-high-risk (BI-RADS 4B and above), 21.1% (16/76) MCs were assessed as low-risk (BI-RADS category 4 A), for which the malignancy likelihood is exceptionally low. By comparing the ultrasonographic features of MCs in the low-risk and medium-high-risk groups, we confirmed that the majority (81.2%) of MCs in the low-risk group were oval shape. Lam et al. [8] reported that the ratio of oval shape masses on US was 40.6% for all MCs, 47.4% for PMCs and 30.8% for MMCs, while Kaoku et al. [15] reported that only 9.1% of PMCs were oval shape. In our research, an oval shape mass on US accounted for 32.9% (25/76) of all MCs, 34.7% of PMCs and 29.6% of MMCs. Apparently, the proportion of oval masses in PMCs was higher than in MMCs, but there was no significant difference between the two in the current study and in previous studies. An oval shape is an ultrasonographic feature of benign breast masses, which may be suggested for follow-up instead of biopsy [10, 11, 18, 19]. On US, if the shape of MC is oval, its risk category may be underestimated. In addition to shape, there were also statistically significant differences in margin features between the low-risk group and the medium-high-risk group. However, although the percentage of MCs with a circumscribed margin in the low-risk group was higher than that in the medium-high-risk group, the majority (75%) of MCs in the low-risk group had not circumscribed margins, which is an ultrasonographic feature of malignancy [6, 7, 10, 11, 19]. Overall, oval shape was the main reason why MCs were underestimated as low-risk which typically suggests a benign mass.

Fibroadenomas are the most common benign tumours of the breast [20]. Compared with patients with FA which is commonly found in young women (typically in 20 to 30 years old) [21], patients with MC in this study were older, with a median age 47 years, which is younger than the 51 to 71 years previously reported [22, 23].

Regarding ultrasonographic features, most FAs are oval or round, well circumscribed, hypoechoic with the long axis parallel to the skin surface and have normal or increased posterior echogenicity [24], which are almost consistent with our study findings. By comparing the other features of MC and FA both with an oval shape, we found that the margin features, internal echo patterns and posterior echo features were helpful for their differential diagnosis. Among internal echo pattern, margin and posterior features, the internal echo pattern is usually the first feature to attract the attention of ultrasound physicians. Hypoechogenicity is the most common type of echo in both typical benign and typical malignant masses [6, 7, 12, 19].

Compared with that of the FAs, the internal echo of most MCs was non-hypoechoic which included heterogeneous, isoechoic, or complex cystic and solid, which may be related to the complex tissue composition of MCs. MCs are composed of large amounts of extracellular mucin, stroma, and clusters of cancer cells. Kaoku et al [15] reported that the internal echo of MC varied with the proportions of the different components, particularly, the proportion of stroma tended to increase as the internal echogenicity increased.

Regarding the difference in the margin features of MCs and FAs, unlike the circumscribed margins of most FAs (84%), the margins of most MCs (84%) were not circumscribed in our study. Previous studies have reported that the percentage of MC with not circumscribed margins was between 23.5% and 95.0% [8, 9, 13, 14, 16]. The size of this range may be related to the different number of patients included in the studies. Compared with previous studies with fewer cases or only including PMCs, our study is more convincing, because it includes the largest number of patients and included two pathologic types, PMC and MMC. The not circumscribed margins of MC are related to the aggressive growth pattern of malignant masses [11, 19]. Furthermore, the posterior enhancement was another common ultrasonographic feature of MC. This is probably due to the transmission of the ultrasound beam through large amounts of extracellular mucin in MC [8].

This study had some limitations. First, our study only included commonly used ultrasonographic features. US elastography was not extensively used in our study, although it was added to the BI-RADS for US. Second, the advantages of artificial intelligence in mucinous carcinoma diagnosis are becoming increasingly prominent [25, 26]. However, we had a small number of patients. We will collect more cases and attempt to use artificial intelligence to reduce the underestimation of mucinous carcinoma with an oval shape. Finally, this was a single-institution retrospective study. Multicentre studies with greater numbers of patients are needed to confirm our findings.

## Conclusion

In this study, MCs with an oval shape on US may be underestimated as low-risk. If an older woman presents with an oval breast mass with non-hypoechoic patterns, a posterior echo enhancement and not circumscribed margins, the possibility of MC should be considered, and active biopsy should be recommended.

## Data Availability

Data can be obtained with appropriate reason by request to the corresponding author through email gchang@mail.sysu.edu.cn.

## Abbreviations

BI-RADS: Breast Imaging Reporting and Data System
FA: Fibroadenoma
MC: Mucinous carcinoma
PMC: Pure mucinous carcinoma
MMC: Mixed mucinous carcinoma
US: Ultrasonography
